# Survey data of household perceptions of drought, mitigation and adaptation practices in East Nusa Tenggara, Indonesia

**DOI:** 10.1016/j.dib.2019.103944

**Published:** 2019-04-20

**Authors:** Heri Kuswanto, Fausania Hibatullah, Eddy Setiadi Soedjono, Ferry Efendi

**Affiliations:** a Department of Statistics and Center for Earth, Disaster and Climate Change-Institut Teknologi Sepuluh Nopember (ITS), Indonesia; b Department of Statistics, Institut Teknologi Sepuluh Nopember (ITS), Indonesia; c Department of Environmental Engineering, Institut Teknologi Sepuluh Nopember (ITS), Indonesia; d Faculty of Nursing, Airlangga University, Indonesia

**Keywords:** Drought, Perception, Risk

## Abstract

A comprehensive and detailed description of household survey data that were collected in East Nusa Tenggara, Indonesia in 2018 is provided in this article. The survey was conducted using a structured questionnaire administered among 300 households in East Nusa Tenggara as one of the regions experiencing severe drought for more than a decade. The information about perceptions of drought and mitigation and adaptation strategies was collected from the head of household or household member. The survey comprises comprehensive information about household socio-demographic characteristics, household resources, agricultural activities, knowledge and perceptions of drought, experience with drought and adaptation strategy, mitigation of the impact of drought, future drought and the participation of women in decision making. The data are provided with this article.

**Table:** Specifications Table

Subject area	Social and behavioural science
More specific subject area	Adaptation and mitigation of drought
Type of data	Figure, table, string, categorical and numerical variables
How data was acquired	Household surveys through face-to-face interviews (structured questionnaire). The questionnaire can be found online at: https://dataverse.harvard.edu/dataset.xhtml?persistentId=doi%3A10.7910%2FDVN%2FLGYJM3
Data format	Raw data and descriptive statistics.
Experimental factors	Sample consisted of 300 households selected randomly from seven districts in East Nusa Tenggara, Indonesia.
Experimental features	Variables related to Drought Cycle Management
Data source location	300 households distributed in Kupang, South Timor Tengah, North Timor Tengah, Ende, Nagekeo, East Flores and Lembata districts of East Nusa Tenggara, Indonesia.
Data accessibility	The data have been made available online at: https://dataverse.harvard.edu/dataset.xhtml?persistentId=doi%3A10.7910%2FDVN%2FLGYJM3
Related research article	P. Udmale, Y. Ichikawa, S. Manandar, H. Ishidaira, A.S. Kiem, Farmers' perception of drought impacts, local adaptation and administrative mitigation measures in Maharashtra State, India, International Journal of Disaster Risk Reduction, 10, 2014, 250–269 [1].

**Table:** 

**Value of the Data** • The dataset can be used to understand households' adaptation and mitigation strategies in relation to drought as part of the Drought Cycle Management model. • The dataset provides a significant contribution to capturing information about households' need related to the weather or seasonal forecasts, as well as the factors influencing the use of the forecast. This analysis could support a better strategy for policy makers to increase awareness, as well as households' acceptance of forecasts (technology acceptance). • The dataset contains key variables to estimate the impact of drought related to the socio-demographic characteristics and perceptions of drought. It can be used as the basis of formulating strategies to minimise the risk of drought. • The dataset captures information about livestock and crop management during drought periods as well as crop and animal loss. It can be used to formulate policy recommendations on the best management to minimise the risk of crop and animal loss.

## 1 Data

**Fig. 1:**
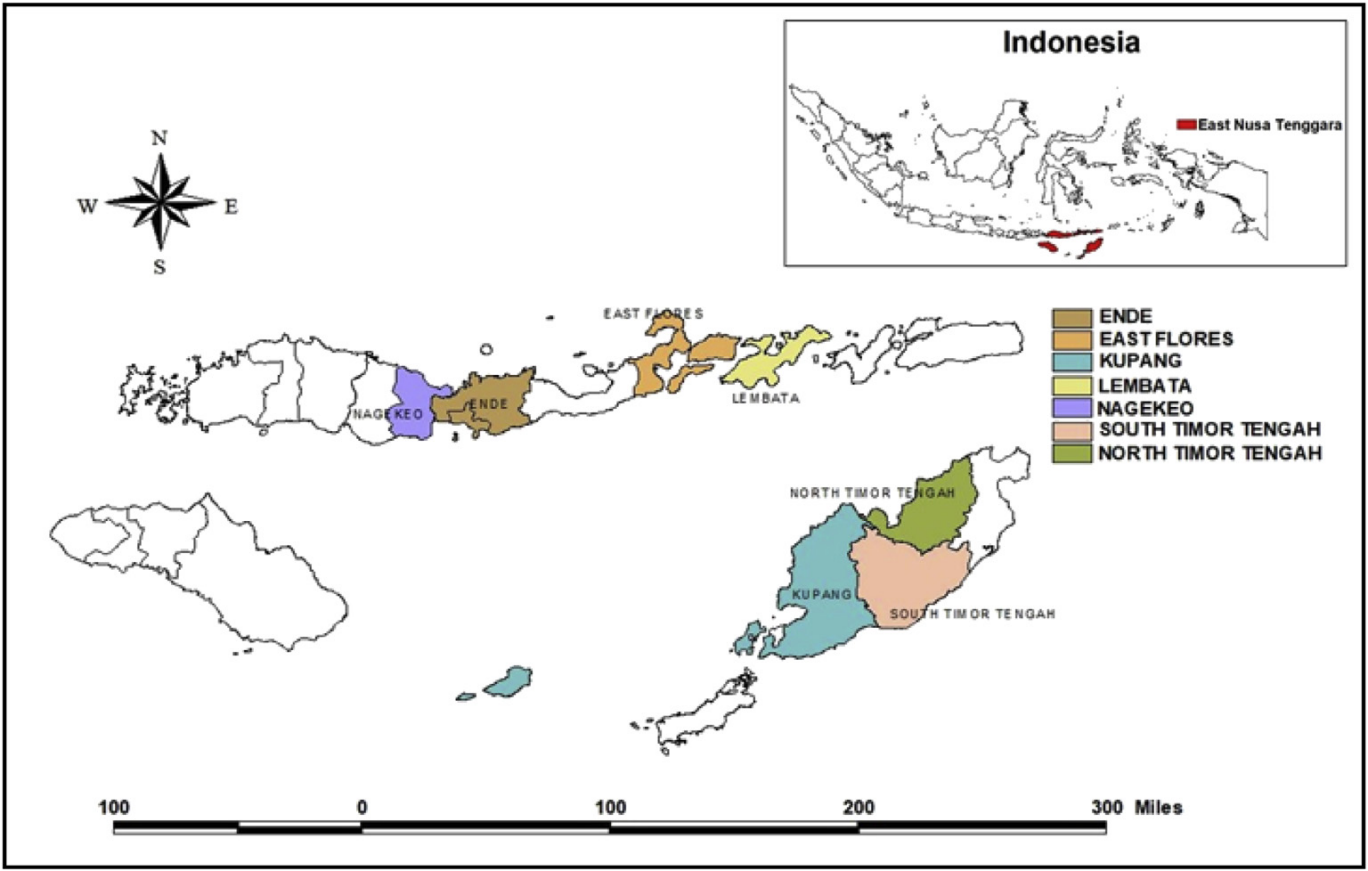
Geographical location of the household survey.

The dataset consist of 300 responses collected from the household survey conducted in seven district in East Nusa tenggara (see Fig. 1). The survey was conducted within a three-month period (June to August 2018) and is part of the project assessing the implementation of the Drought Cycle Management (DCM) model. East Nusa tenggara is the region most vulnerable to drought in Indonesia [2], [3], [4]. The questionnaire was developed following the work of [5] with some modifications. The dataset comprises information about household socio-demographic characteristics (e.g. gender, age, marital status, monthly income, education, etc.) as well as drought related issues, i.e. household resources (e.g., water source, household income, farming land ownership, etc.), agricultural activities (i.e., type of crops and livestock, water source for crop and livestock management, problems encountered during drought, etc.), knowledge and perception about drought including the perception of weather forecasts, experience with drought (e.g., crop and animal loss) and adaptation strategies, mitigation of drought impact, perception of future drought and the participation of women in decision making.

Table 1 provides several key pieces of information about the socio-demographic characteristics of the respondent and household.

**Table 1:** Household socio-demographic characteristics.

Variable	Category	Count	Percentage
Gender	Male	183	61%
	Female	117	39%
Household Head	Yes	198	66%
	No	102	34%
Average age	Respondent	45	
	Household head	49	
	Wife of household head	44	
Level of education of household head	Never went to school	22	7.3%
	Primary school	191	63.7%
	Junior/Senior high school	84	28.0%
	University	3	1.0%
Monthly income	< IDR 500000	208	70.03%
	IDR 500000 - IDR 2000000	82	27.61%
	IDR 2000000 – IDR 4000000	7	2.36%
	> IDR 4000000	0	0.00%
Length of stay	<1 year	10	3.33%
	1–5 years	7	2.33%
	5–10 years	10	3.33%
	>10 years	273	91.00%
House ownership	Own	286	95.33%
	Rent	1	0.33%
	Other	13	4.33%

## 2 Experimental design, materials and methods

The sampling design applied to acquire the data is a two-stage and stratified random sampling as in Ref. [6]. The total number of respondents to be surveyed is 300 households in East Nusa Tenggara. The sampling was designed to ensure that the collected sample is representative of the target population in the region. The first stage selects randomly seven districts out of 22 districts in East Nusa Tenggara. The second stage includes selecting a total of 300 households from the selected districts. The number of surveyed households in a district is allocated proportionally to the total number of households in the corresponding district. Households were chosen randomly from the list of all households' name in the selected district. Face-to-face interviews using a paper questionnaire were conducted to collect the data. The main target as the respondent was the head of household. Nevertheless, due to the farming activities during the day, it was only possible to interview 198 household heads, while the other 102 questionnaires were undertaken with another household member who had sufficient information on the implemented drought adaptation and mitigation strategies. The fieldwork was carried out by teams involving trained local surveyors to ensure that the respondents understand the asked questions.

The collected data were entered in excell. Furthermore, the raw data were refined by correcting any inconsistent inputs. Moreover, outliers and missing responses were cleaned from the data. The descriptive statistics in this paper were performed by using the Statistical Package for Social Sciences (SPSS) software.
