# Supplementary material for: Survey data of household perceptions of drought, mitigation and adaptation practices in East Nusa Tenggara, Indonesia
Source: Data Brief. 2019 Apr 20;24:103944. doi: 10.1016/j.dib.2019.103944 (PMC6502731; doi:10.1016/j.dib.2019.103944)
Supplement: Multimedia component 3 [file mmc3.pdf]

Part I	Household Demographic Characteristics		
Q1.1	Area of residence		
Q1.2	Geographical location	Province	
		District	
		Sub-district	
		Village	
Q1.3	Sex of respondent	Male	
		Female	
Q1.4	Are you the head of household (nuclear family)?	Yes	
		No	
Q1.4A	If not the head, what is the sex of the head of household?	Male	
		Female	
Q1.4B	If not the head, what is your relation with the head of the household?	Spouse	
		Son	
		Daughter	
		Relative	
		Other	
Q1.5	Marital status	Single	
		Married-Monogamous	
		Married-Polygamous	
		Divorced/separated	
		Widowed	
Q1.6	Age in years	Age of respondent	
		household head	
		spouse of household head	
Q1.7	Ethnicity (Tribe)		
Q1.8	Level of education of household head	None	
		Primary school	
		Secondary school	
		Post secondary	

Q1.8A	Level of education of spouse of household head	None		
		Primary School		
		Secondary School		
		Post secondary		
Q1.9	Household size		Male	Female
		0 - 5 years		
		6 - 18 years		
		18-55 years		
		> 55 years		
Q1.10	Number of children in schooling		Boys	Girls
		Primary (Std 1-8)		
		Secondary		
		Post secondary		
Q1.11	Number of economically active members living in household		Male	Female
		Unpaid family worker		
		Self-employed/small-scale business		
		Wage Employment		
		Other (specify)		
Q1.12	Number of economically inactive members living in the household		Male	Female
		Too young		
		Too old		
		Sick		
		Disabled		
		Other (specify)		
Q1.13	Number of members living outside sub-location (migrated, working etc)		Male	Female
		Within the district		
		Within the Province		
		Elsewhere in Indonesia		
		Outside Indonesia		
		< Rp. 500.000		

Q1.14	What is the total annual income of your household?	Rp.500.000 - Rp 2.000.000	
		Rp. 2.000.000 - 4.000.000	
		> Rp. 4.000.000	
Q1.15	How long have you lived in this area?	< 1 year	
		1-5 years	
		5-10 years	
		> 10 years	
Q1.16	Do you own or rent your home?	Own	
		Rent	
		Other (specify)	

Part II	***Household Resources***		
Q2.1	Type of house	Semi-permanent (mud wall/tin roof)	
		Semi-permanent (mud wall/grass roof)	
		Temporary (grass wall and roof)	
Q2.2	Household assets (more than one answer allowed)	Radio	
		Television	
		Bicycle	
		Mobile phone	
		Water tanks	
		Car	
		Computer	
		Internet	
		Refrigerator	
		Improved sources of lighting	
		Cooking fuel	
		Toilet	
		Drinking water	
		Spring	

Q2.3	Water Sources	Water tank	
		Lake	
		River	
		Piped water	
		Public tap	
		Rain water collection	
		Other (specify)	
Q2.4	Household income	Single income source	
		Multiple sources of income	
Q2.4B	What is the key source of livelihood for the household?	Pastoralism	
		Agropastoralism	
		Small scale business	
		Wage employment	
Q2.5	What are the major constraints to your family well-being?	Drought	
		Famine	
		Floods	
		Human diseases	
		Livestock diseases	
		Conflicts/insecurity	
		Poverty	
		Other (specify)	
Q2.6	Do you have access to land to grow crops or graze livestock?	Yes	
		No	
	How many acres of farm land does your household use?	None	
		< 1 acre	
		1-5 acres	
		> 5 acres	
	Ownership of farming land	Individually owned	
		Communally owned	
		Rented	
		Temporary loan	

		Other (specify)		
Q2.7A	Is the water source for the household constant or seasonal?	Constant supply (1) Seasonal (2)		
Q2.7B	Who manages the water source for the household? (Tick where appropriate)	No management		
		Individually owned		
		community		
		Other (specify)		
Q2.8	Do you rely on the crops and livestock to feed your family?	Yes		
		No		

Part III	***Household agricultural activities***			
Q3.1	Do you grow crops?	Yes (Continue to Q3.1A)		
		No (Continue to Q3.2)		
Q3.1A	If an agro-pastoralist, what type of crops do you grow? What was the maximum yield over the past 5 years assuming normal rains? How much did you sell, if any?		Number Yield (90 kg bags)	Sales over last one year
		Maize		
		Cassava		
		Sweet potato		
		Rice		
		Sorghum		
		Ground nuts		
		Beans		
		Other (specify)		
Q3.1B	What problems do you usually encounter with regard to grow the crops? Tick where appropriate	Crop failure due to pests		
		Water and pasture shortages		
		Lack of market		
		Conflicts/insecurity		
		Other (Specify)		
		I don't have any problems		
			Number Yield (90 kg bags)	

Q3.1C	Looking at the same types of crops, how much would you be able to harvest with limited rainfall? (limited rainfall should be specified (e.g.,75% of normal, 50% of normal or if the rainy season is delayed)	Rice	
		Maize	
		Sorghum	
		Beans	
		Other (specify)	
Q3.1D	Is the land under irrigation? If no, indicate source of water?	Yes	
		No	
Q3.1E	What is the main source of water for the crops? Tick where appropriate	River/spring/stream	
		Water pans or dams	
		Wells/Boreholes	
		Rock catchment	
		Piped water	
		Other (Specify)	
Q3.1F	Is the water source for the crops constant or seasonal?	Constant supply (1) Seasonal (2)	
Q3.1G	Who manages the water source for the crops? (Tick where appropriate)	No management	
		Individually owned	
		community	
		Other (specify)	
Q3.1H	How do you contribute to the maintenance of the water source for the crops? (Tick where appropriate)	Does not contribute anything	
		Contributes set fee	
		Contributes in case of a break down	
		Contributes manual labour when required	
Q3.2	Do you own livestock?	Yes (Continue to Q3.2A)	
		No (Continue to Q3.3)	
Q3.2A	What types of livestock do you keep? On average (over the past 5 years), what is the size of your stock?		Number
		Cattle – Bulls	
		Cows	
		Sheep	
		Goats	

		Poultry	
		Others	
Q3.2B	What problems do you usually encounter with regard to livestock keeping? Tick where appropriate	Livestock diseases	
		Water and pasture shortages	
		Lack of market	
		Livestock rustling	
		Conflicts/insecurity	
		Other (Specify)	
		I don't have any problems	
Q3.2C	What measures have you put in place to address the above mentioned problems? Tick where appropriate	Migration in search of pasture and water	
		Restocking through traditional systems	
		Use of traditional herbal treatment	
		Accessing livestock veterinary services	
		Sale of animals during drought	
		Other (Specify)	
Q3.2D	What is the main source of water for the livestock? Tick where appropriate	River/spring/stream	
		Water pans or dams	
		Wells/Boreholes	
		Rock catchment	
		Piped water	
		Other (Specify)	
Q3.2E	Is the water source for the livestock constant or seasonal?	Constant supply (1) Seasonal (2)	
Q3.2F	Who manages the water source for the livestock? (Tick where appropriate)	No management	
		Individually owned	
		community	
		Other (specify)	
	[illegible]	Does not contribute anything	
		Contributes set fee	

Q3.2G	How do you contribute to the maintenance of the water source for the livestock? (Tick where appropriate)	Contributes in case of a break down	
		Contributes manual labour when required	
Q3.3	If employed or running a business, on average, how much income do you get in a month?		

Part IV	*Knowledge and perception of drought*		
Q4.1	Do you understand about drought?	Yes	
		No	
Q4.1A	What is your understanding of drought?		
Q4.2	What causes droughts? Tick where appropriate	Amount of rainfall	
		Timing of rainfall	
		Duration of rainfall	
		Change in soil type	
		Change in vegetation (e.g.deforestation)	
		All of that above	
		I don't know	
Q4.3	What are the effects of drought? Tick where appropriate (Specify recent or ongoing drought)	Drying of water sources	
		Famine	
		Crop failures	
		Loss of livestock	
		Poor health of humans	
		Poor health of animals	
		Increase in food prices	

		Decline in livestock prices	
		Other (specify)	
Q4.4	Do you use weather / seasonal forecasts?	Yes	
		No	
Q4.5	How do you get the information on weather / seasonal forecasts?	Radio/TV	
		Extension agent	
		Word of mouth	
		Traditional sources	
		Other (specify)	
Q4.6	How do you know what the weather may be like during dry or rainy season?		
Q4.7	How do you respond to weather/seasonal forecasts?	Change your normal agricultural practices	
		Try to get more information	
		Don't do anything differently	
		Others	
Q4.8	Do you use information about weather/seasonal forecast issued by government (BMKG or others)?		
Q4.9	What do you think about accuracy of wheather/seasonal forecast so far?		

Part V	***Drought impacts and experience***		
Q5.1A	How many animals have you sold in the last year?		Number
		Cattle – Bulls	
		Cows	
		Sheep	
		Goats	

		Poultry		
		Others		
Q5.1B	Why did you sell the animals?	Income generation		
		Sale during drought		
		Restocking		
Q5.1C	Which members of the household were involved in the decision to sell the animals? [RESPONDENT GIVES NAMES OF HOUSEHOLD MEMBERS, ENUMERATOR THEN CODES THEM BY GENDER]	Head of the household alone		
		Other male members of the household		
		Joint decision between various male and female members of the household		
		Other female members of the household		
Q5.1D	How many animals did you receive/give as gifts last year?		Receive	Give
		Cattle – Bulls		
		Cows		
		Sheep		
		Goats		
		Poultry		
		Others		
Q5.1E	How many animals did you lose due to disease last year?		Number	
		Cattle – Bulls		
		Cows		
		Sheep		
		Goats		
		Poultry		
		Others		
Q5.1F	How many animals did you lose due to drought last year?		Number	
		Cattle – Bulls		
		Cows		
		Sheep		
		Goats		
		Poultry		

		Others	
Q5.2A	How many crops did you lose due to pests last year?		Number Yield (90 kg bags)
		Maize	
		Cassava	
		Sweet potato	
		Rice	
		Sorghum	
		Ground nuts	
		Beans	
		Others	
Q5.2B	How many crops did you lose due to drought last year?		Number Yield (90 kg bags)
		Maize	
		Cassava	
		Sweet potato	
		Rice	
		Sorghum	
		Ground nuts	
		Beans	
		Others	
Q5.3	How does drought impact your livelihood?		

Part VI	***Preparedness and Coping with drought impacts***		
	Have you experienced drought?	Yes	
		No	
		spatial diversification of fields	
		livestock / farming diversification	

Q6.1	What measures do you put in place in preparation to safeguard yourself against a coming drought?	livestock /farming management adjustments (changes in feed,	
		water, grazing land use)	
		access to extension services for knowledge of livestock farming during droughts	
		livestock insurance	
		income diversification	
	What measures do you put in place to safeguard yourself during drought?	use of savings	
		claim insurance	
		migrate	
		sell livestock	
		ask family for help	
		other (specify)	
Q6.1B	Which members of the household are involved in deciding which measures to put in place when safeguarding against a coming or during drought?	Head of the household alone	
		Other male members of the household	
		Joint decision between various male and female members of the household	
		Other female members of the household	
Q6.2	Considering the source of livelihood in 2.1, do you seek additional sources of income when anticipating drought?<br><br>Yes (1) No (2)		
	If yes, which are these additional sources of income?	Sale of assets	1
		Seeking employment	2
		Starting a business	3
Q6.3	Do you reserve water for use during the drought? Yes (1) No (2)		
Q6.4	Do you reserve pasture for use during the drought? Yes (1) No (2)		
		Relief supplies	1
		Use reserve	2

Q6.5	What is the main source of water and pasture/hay for the household during the drought season? (more than one answer allowed)	Buy hay from suppliers	3
		Take livestock to rented grazing land	4
		Migrate livestock within district	5
		Migrate livestock outside district	6
		Other (specify)	7
Q6.6	In case of drought, which animals (plant) would you rather have?		Yes (1) /No (2)
		Cattle – Bulls (Rice)	
		Cows (Maize)	
		Sheep (Shorgum)	
		Goats (Beans)	
		Poultry (Others)	
		Others	
Q6.7	During drought, what adjustments do you make in terms of food consumption? How do you cope with food shortages?	Depleting food and cash savings	1
		Earning more wage income	2
		Credit/ Borrowing	3
		Liquidating productive assets (livestock, land, farm tools and building)	4
		Liquidating other assets (gold, ornaments, and jewellery)	5
		Household food consumption adjustments	6
		Relying on charity	7
		Use of social network	8
		Permanent or seasonal migration	9
		Village-level institutions	10
		Off-farm employment	11
		Household expenditure	

		adjustments (clothes, education and health)	12	
		Relying on publicly sponsored relief programs	13	
Q6.8	What are the practises for using and conserving natural resources such as pasture, forests, water etc?	Having drought reserve grazing	1	
		Protection of specific plant species or areas	2	
		Having individual or communal user rights for water/grazing points	3	
		Other (specify)	4	
Q6.9	If the drought was severe, would you migrate your family out of the pastoral livelihood?			
	Yes (1) No (2)			
Q6.9A	Which members of the household would need to be involved in the decision to migrate the family out of the pastoral livelihood?	Head of the household alone		
		Other male members of the household		
		Joint decision between various male and female members of the household		
		Other female members of the household		
Q6.10	What livelihood options do you have, apart from pastoralism?	Farming	1	
		Wage employment	2	
		Small scale business	3	
Q6.11	Did you sell any other household assets?		Yes (1) / No (2)	If yes, how much?
		Radio		
		Television		
		Bicycle		
		Mobile phone		

		Water tanks		
		Donkey cart		
		Jewellery		
		Farm implement		
		Other (specify)		
Q6.11A	What was the main reason for selling the assets?	Buying food	1	
		Buying clothing	2	
		Paying for healthcare	3	
		Paying for the farm	4	
		Transport expenses	5	
		To fund cultural ceremonies e.g. marriages	6	
		Other (specify)	7	
Q6.11B	Which members of the household were involved in the decision to sell the assets?	Head of the household alone		
		Other male members of the household		
		Joint decision between various male and female members of the household		
		Other female members of the household		
Q6.12	Did you borrow any money in the last one year? If so, how much?		Yes (1)/No (2)	How much?
		Bank		
		Co-operative/SACCO		
		Family/friends		
		Other (specify)		
Q6.13	What was the major reason for borrowing the money?	Buying food	1	
		Buying clothing	2	
		Paying for healthcare	3	
		Paying for the farm	4	
		Transport expenses	5	
		To fund cultural ceremonies e.g. marriages	6	

		Other (specify)	7
Q6.14	What type of support do you get from the following: (Circle where appropriate)	Government agencies	(1) Information (2) Provision of social services (3) Emergency aid (4) Development aid (5) Financial assistance i.e. loans and grants (6) Advocacy assistance
		NGOs	(1) Information (2) Provision of social services (3) Emergency aid (4) Development aid (5) Financial assistance i.e. loans and grants (6) Advocacy assistance
		Religious organizations	(1) Information (2) Provision of social services (3) Emergency aid (4) Development aid (5) Financial assistance i.e. loans and grants (6) Advocacy assistance
			(1) Information (2) Provision of social services (3)

		Family/friends	Emergency aid (4) Development aid (5) Financial assistance i.e. loans and grants (6) Advocacy assistance

Part VII	***Future Droughts***		
Q7.1	Would you use weather forecast in the future?	Yes	
		No	
Q7.1B	If yes, how would you prefer to receive the weather and seasonal forecast?	TV	
		Community leaders	
		Extension Agents	
		Text messages on cell phone	
		Mail	
		Radio	
		Other (Specify)	
Q7.2	When would the weather information be most useful?		

Part VIII	***Woman Participation in decision making***		
	[Should be asked of a WOMAN in the household. If the respondent for parts I-IV was a woman, simply continue the survey. If the respondent thus far was a man, say that the next portion of the survey refers to women in the household, and ask to speak with an adult female member of the household.]		
		I can make the decision on my own	
		I must consult with other female members of the household	

Q8.1	When a child is sick and you want to take them to the doctor, can you take action on your own or must you consult with other members of the household?	I must consult with my husband (or other male member of the family) and we will discuss it and make the decision together	
		I must receive permission from my husband (or other male member of the family), and he alone will make the final decision	
Q8.2	When you need to buy medicine for yourself, can you take action on your own or must you consult with other members of the household?	I can make the decision on my own	
		I must consult with other female members of the household	
		I must consult with my husband (or other male member of the family) and we will discuss it and make the decision together	
		I must receive permission from my husband (or other male member of the family), and he alone will make the final decision	
Q8.3	When you wish to make a trip to visit your family or friends, can you take action on your own or must you consult with other members of the household?	I can make the decision on my own	
		I must consult with other female members of the household	
		I must consult with my husband (or other male member of the family) and we will discuss it and make the decision together	
		I must receive permission from my husband (or other male member of the family), and he alone will make the final decision	

Q8.4	When you wish to make small household purchases that may be needed on a daily basis, such as (INSERT LOCALLY APPROPRIATE EXAMPLE HERE), can you take action on your own or must you consult with other members of the household?	I can make the decision on my own	
		I must consult with other female members of the household	
		I must consult with my husband (or other male member of the family) and we will discuss it and make the decision together	
		I must receive permission from my husband (or other male member of the family), and he alone will make the final decision	
Q8.5	When you wish to make a large purchase, such as (INSERT LOCALLY APPROPRIATE EXAMPLE), can you take action on your own or must you consult with other members of the household?	I can make the decision on my own	
		I must consult with other female members of the household	
		I must consult with my husband (or other male member of the family) and we will discuss it and make the decision together	
		I must receive permission from my husband (or other male member of the family), and he alone will make the final decision	
Q8.6	When you wish to take an action to help safeguard the household against a coming drought, for example, related to livestock/farming management, can you take action on your own or must you consult with other members of the household?	I can make the decision on my own	
		I must consult with other female members of the household	
		I must consult with my husband (or other male member of the family) and we will discuss it and make the decision together	

		I must receive permission from my husband (or other male member of the family), and he alone will make the final decision	
Q8.7	If you wish to take an action to diversity the household income, for example by seeking employment or starting a business, can you take action on your own or must you consult with other members of the household?	I can make the decision on my own	
		I must consult with other female members of the household	
		I must consult with my husband (or other male member of the family) and we will discuss it and make the decision together	
		I must receive permission from my husband (or other male member of the family), and he alone will make the final decision	
Q8.8	If you wish to take an action to increase household savings, for example by cutting expenses for (INSERT LOCALLY APPROPRAITE EXAMPLE), can you take action on your own or must you consult with other members of the household?	I can make the decision on my own	
		I must consult with other female members of the household	
		I must consult with my husband (or other male member of the family) and we will discuss it and make the decision together	
		I must receive permission from my husband (or other male member of the family), and he alone will make the final decision	
Q8.9	Thinking about decisions made during the most recent drought preparations, did you feel that other members of the household listened to your thoughts, ideas and opinions?	No	
		Yes	
Q8.10	Thinking about decisions made during the most recent drought preparations, did you agree with the decisions made by the household?	No	
		Yes	
